# Parallel identification of novel antimicrobial peptide sequences from multiple anuran species by targeted DNA sequencing

**DOI:** 10.1186/s12864-018-5225-5

**Published:** 2018-11-20

**Authors:** Tomislav Rončević, Marco Gerdol, Francesca Spazzali, Fiorella Florian, Stjepan Mekinić, Alessandro Tossi, Alberto Pallavicini

**Affiliations:** 1 Department of Physics, Faculty of Science, University of Split, 21000 Split, Croatia; 2 Department of Life Sciences, University of Trieste, 34127 Trieste, Italy; 3 Public Institution for the Management of Protected Areas in the County of Split and Dalmatia – “Sea and karst”, 21000 Split, Croatia

**Keywords:** Antimicrobial peptides, Anura, Innate immunity, Parallel identification, Signal peptide region

## Abstract

**Background:**

Antimicrobial peptides (AMPs) are multifunctional effector molecules that often combine direct antimicrobial activities with signaling or immunomodulatory functions. The skin secretions of anurans contain a variety of such bioactive peptides. The identification of AMPs from frog species often requires sacrificing several specimens to obtain small quantities of crude peptides, followed by activity based fractionation to identify the active principles.

**Results:**

We report an efficient alternative approach to selectively amplify AMP-coding transcripts from very small amounts of tissue samples, based on RNA extraction and cDNA synthesis, followed by PCR amplification and high-throughput sequencing of size-selected amplicons. This protocol exploits the highly conserved signal peptide region of the AMP precursors from Ranidae, Hylidae and Bombinatoridae for the design of family-specific, forward degenerate primers, coupled with a reverse primer targeting the mRNA poly-A tail.

**Conclusions:**

Analysis of the assembled sequencing output allowed to identify more than a hundred full-length mature peptides, mostly from Ranidae species, including several novel potential AMPs for functional characterization. This (i) confirms the effectiveness of the experimental approach and indicates points for protocol optimization to account for particular cases, and (ii) encourages the application of the same methodology to other multigenic AMP families, also from other genera, sharing common features as in anuran AMPs.

**Electronic supplementary material:**

The online version of this article (10.1186/s12864-018-5225-5) contains supplementary material, which is available to authorized users.

## Background

AMPs are endogenous antibiotics present in all organisms, with a direct antimicrobial activity towards pathogens, often also showing immunomodulatory properties, and a regulated gene expression to facilitate and modulate immune responses [1, 2]. The skin secretions of many anurans contain a variety of bioactive peptides encoded by multigenic families [3] that often exhibit antibacterial activity towards multidrug resistant microbial isolates [4]. AMPs have been identified in all anuran families of the phylogenetically more ancient suborder of Archaeobatrachia including Leiopelmatidae, Alytidae, Bombinatoridae and Pipidae families. In Neobatrachia, AMPs have been identified in Dicroglossidae, Hylidae, Hyperoliidae, Leptodactylidae, Myobatrachidae and, in particular, in Ranidae [5]. The latter family consists of wide-ranging frog species distributed worldwide, except for the polar regions, in which 14 different AMP classes have been identified to date based on the molecular characteristics of the peptides they contain. Ranid frogs, in general, are well known to synthesize and secrete multiple active AMPs (at least 22 are reported in the skin secretions of *Rana palustris*) with rare exceptions such as in *Rana sylvatica,* in which only one antimicrobial peptide has been isolated to date [6]. It is worth noting that production of antimicrobial peptides may be influenced by hormonal and/or environmental factors [7–9], which can hinder the identification of AMPs under certain experimental conditions.

Identifying novel anuran peptides normally requires handling several individuals, which are either sacrificed or held in captivity and treated with electric shocks/norepinephrine to obtain small amounts of crude peptide. This is followed by several rounds of purification using different precipitation and chromatographic techniques combined with activity testing of fractions to identify the active principles [10]. This approach however raises problems of animal protection and nature preservation. The International Union for Conservation of Nature (IUCN) reports that 1276 amphibian species worldwide are endangered or critically endangered with 38 having become extinct [11]. In this context, the search for a more efficient and less invasive method that requires minimum amounts of biological samples is highly desirable, such as isolation, amplification and sequencing of the nucleotide sequences coding for the AMPs. Although a few alternative approaches based on the screening of available transcriptomic data have been attempted [12], they did not implement the use of degenerate primers designed on the most conserved regions of AMP precursors [13]. This has resulted in the discovery of a limited number of novel AMPs and has never been applied for large-scale multispecies screening.

We have developed a potentially faster, less invasive and more efficient approach based on the selective amplification and subsequent sequencing of transcripts encoding for antimicrobial peptides, starting from very small amounts of tissue. This method however requires accurate primer design to capture the diversity of AMPs. In general, anuran antimicrobial peptide precursors consist of a highly conserved signal sequence, a negatively charged propeptide and a hypervariable cationic mature region [14–16]. Data on anuran signal sequences pertaining to different families and species are available in a dedicated database, DADP [17]. In many cases, the sequences present in this database were validated by biochemical methods, which also confirmed biological activity. Using this information and combining it with a method based on the 3’-RACE (rapid amplification of cDNA ends) protocol [18] we have developed a methodology for simultaneous identification of novel antimicrobial peptide sequences from multiple anuran species. To this purpose, total RNA was extracted from eight different frog species belonging to three anuran families. cDNA libraries were prepared utilizing a reverse primer based on the mRNA poly-A tail and forward degenerate primers designed based on highly conserved signal regions of the peptide precursors. These were used for selective amplification of the target AMP cDNAs, and the resulting amplicons then size-selected and subjected to Ion Torrent long-read high-throughput sequencing. We present data on the effectiveness of this method in identifying AMPs, including several known sequences and a number of novel sequences, some belonging to known classes. We also indicate deficiencies, discuss the most likely causes and indicate how to possibly overcome them.

## Methods

### Tissue sampling and RNA extraction

**Fig. 1:**
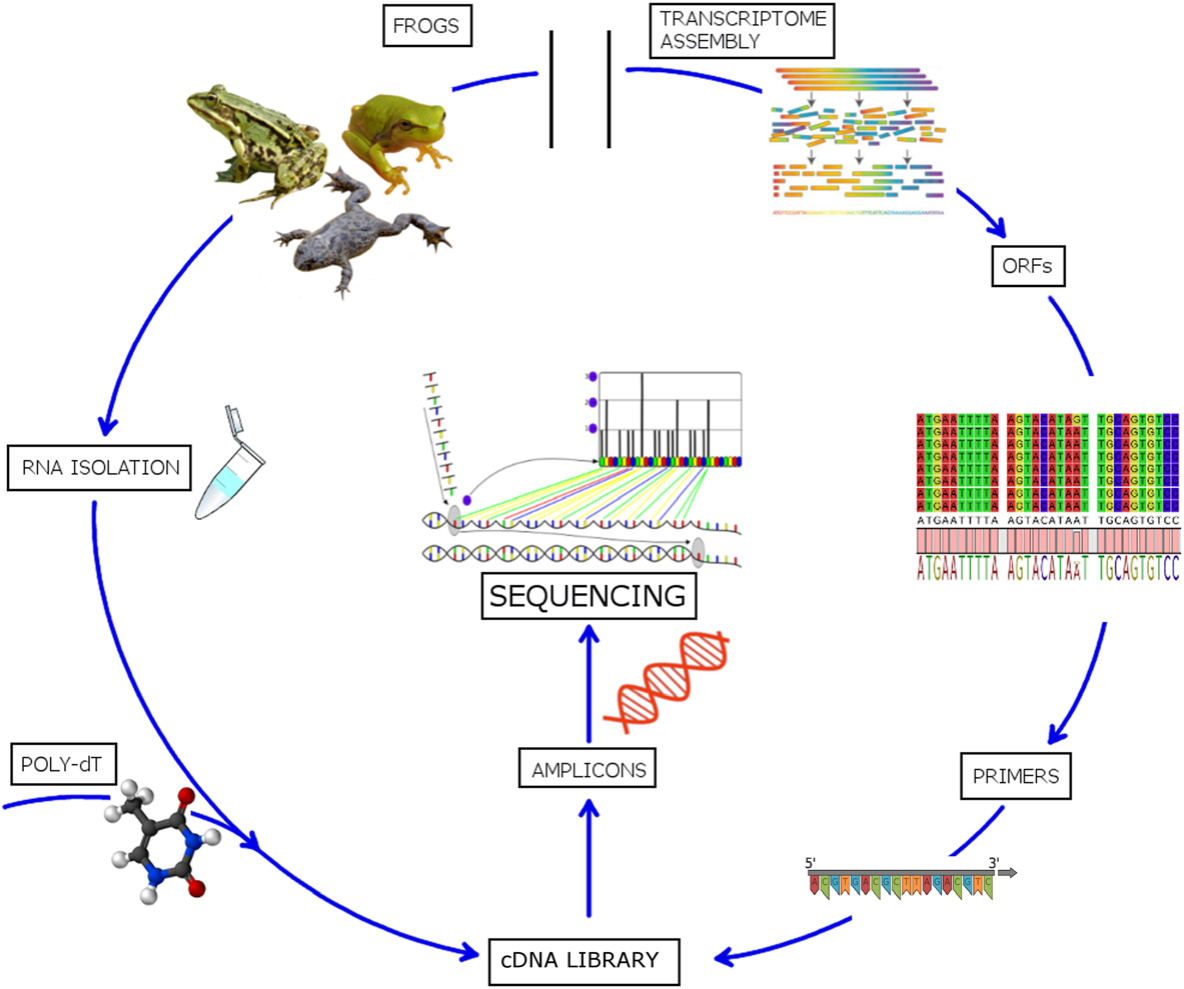
Schematic representation of the targeted DNA sequencing method

One specimen belonging to each of eight different species from Ranidae, Hylidae and Bombinatoridae family (see Additional file 1) was collected in the Croatian wild during March and April 2017. Frogs were sampled in accordance with applicable EU and Croatian legislation governing animal experimentation (Directive 2010/63/EU and NN 55/2013) and necessary permits were obtained from Croatian Ministry of Environmental and Nature Preservation. All animals were sacrificed by exposure to chloroform 24–48 h after capture to ensure a minimal stay in captivity and suffering. Approximately 200 mg of skin tissue was immediately transferred to RNA*later*® buffer (Thermo Fisher Scientific, Waltham, Massachusetts, USA) and stored at − 20 °C according to the manufacturers’ instructions. Total RNA was extracted using the TRIzol protocol (TRIzol® Reagent, Life Technologies, Carlsbad, California, USA) from ~ 50 mg of this tissue, resuspended in RNAase free water, quantified with NanoDrop 2000 (Thermo Fisher Scientific, Waltham, Massachusetts, USA), quality checked using denaturing 1.5% agarose gel electrophoresis, and stored at − 80 °C until further use (see Fig. 1).

### Transcriptome assembly and screening

The available RNAseq data in Sequence Read Archive database [19] was retrieved for 16 anuran species belonging to 3 different families, and assembled with the Trinity 2.4.0 software [20] (see Additional file 2) using default parameters and setting a minimal contig length to 200 nucleotides. Signal sequences pertaining to Class-1 (Ranidae and Hylidae) and Class-3 (Bombinatoridae) families were obtained from DADP database taking into account only peptides with reported bioactivity data [17] (see Additional file 3). The protein sequences from DADP were aligned using Muscle [21] and used to generate Hidden Markov Model profiles with the HMMER 3.1b1 hmmbuild module [22]. Trinity assembled transcripts were translated to all six possible reading frames with EMBOSS transeq [23, 24] and then screened for significant matches using the HMMER 3.1b1 hmmsearch module with an E-value cut off < 0.05. Open reading frames (ORFs) encoding peptides corresponding to positive hits were extracted from each transcriptome with CLC Genomics Workbench 10.1.1 (Qiagen, Hilden, Germany). Incomplete ORFs were also retained.

### Primer design

The nucleotide sequences from Ranidae and Hylidae AMP transcripts obtained as described above were separately aligned and only the regions encoding for the signal peptides were kept for further analysis. Redundancies were removed and the remaining sequences were clustered by similarity with the cd-hit software, using a 0.8 identity threshold (i.e. all signal peptide sequences sharing > 50% sequence identity at the nucleotide level were clustered together) [25]. The same procedure was used for the Bombinatoridae family, but in this case longer highly conserved regions were obtained comprising the propeptide region. The resulting alignments of sequence clusters were used for forward primer design (see Additional file 4). Briefly, the position of forward primers was selected based on the identification of well-conserved 20 nucleotide-long sequence stretches containing a maximum of 3 polymorphic positions, where degenerate nucleotides were inserted (see Table 1). Due to the short length of the signal peptide region of Ranidae and Hylidae AMPs (about 60 nucleotides), the positioning of the forward degenerate primer was not expected to have a significant effect on amplicon size and therefore the maximum allowance of 3 polymorphic positions was aimed at minimizing the chances of non-specific PCR product amplification. In the case of Bombinatoridae, due to the different organization of AMP precursors, the forward primer was designed as close as possible to the 3’end of the propeptide-encoding region, maintaining the maximum limit of three degenerate nucleotides.

**Table 1:** List of primers used for cDNA synthesis and amplification

Target family	Primer	Primer sequence (5′-3′)
Forward primers		
Ranidae^a^	TP1	CAGGACCAGGGTACGGTG**ATGTTCACCTTGAAGRAATC**
Ranidae^a^	TP2	CAGGACCAGGGTACGGTG**TTGGGATYGTCTCCTCATCT**
Ranidae^a^	TP3	CAGGACCAGGGTACGGTG**ATGTTCACCWTGARKAAAYC**
Hylidae^b^	TP4	CAGGACCAGGGTACGGTG**AAGAARTCWCTTYTTCTTGT**
Bombinatoridae^c^	TP5	CAGGACCAGGGTACGGTG**TAGAAGAAGAATCACTGAGG**
Outer PCR *Forward*	A-Uni1	CCATCTCATCCCTGCGTGTCTCCGACTCAG(X)_10_CAGGACCAGGGTACGGTG
PCR *Reverse*	trP1	CCTCTCTATGGGCAGTCGGTGAT

AMP specific sequences are in bold

^a^Species pertaining to Ranidae family: *Pelophylax ridibundus*, *Pelophylax* kl. *esculentus*, *Rana dalmatina*, *Rana arvalis* and *Rana temporaria*

^b^Species pertaining to Hylidae family: *Hyla arborea*

^c^Species pertaining to Bombinatoridae family: *Bombina variegata* and *Bombina bombina*

The reverse primer (5’-CCTCTCTATGGGCAGTCGGTGATTTTTTTTTTTTTTTTTTTT-3′) contained a poly-dT stretch to match the poly-A tail of the mRNA and was used for cDNA synthesis. It also contained a 5′ tail sequence (trP1) which was required for the cDNA amplification protocol and subsequent parallel sequencing. All forward primers (see Table 1**)** were also synthesized with a 5’-CAGGACCAGGGTACGGTG-3′ tail required for multiplex sequencing through attachment of the barcodes in a secondary outer amplification. All primers were synthesized by BMR Genomics (Padova, Italy) (see Fig. 1).

### Library construction and sequencing

First strand cDNA was synthesized from 1 μg of total RNA using the qScript™ Flex cDNA Synthesis Kit (Quanta Biosciences, Gaithersburg, Maryland, USA) according to the manufacturers’ instructions. The reverse transcription reaction was performed at 37 °C for 1 h. The mixture for cDNA amplification contained 0.2 μl of DNA polymerase (5 U/μl), 2.5 μl of 25 mM MgCl_2_, 2.5 μl of 10 × buffer A (KAPA Taq PCR Kit, Kapa Biosystems, Wilmington, Massachusetts, USA) together with 0.5 μl of 10 μM dNTP solution, 0.5 μl of 10 μM specific forward primer solution, 0.5 μl of 10 μM trP1 primer solution and 1 μl of cDNA template in a total volume of 20 μl. The PCR started with an initial denaturation at 95 °C for 2 min, followed by 10 cycles including 10 s at 95 °C, annealing at 45–50 °C for 20 s (ramping 0.5 °C/cycle) and 20 s elongation at 72 °C. For the next 25 cycles the annealing temperature was set to 52 °C for 20 s ending with 5 min final elongation at 72 °C (MJ Research PTC-200 Gradient Thermal Cycler, Marshall Scientific, Hampton, New Hampshire, USA).

Outer PCR was performed to attach the barcodes (a sample-specific 10 nucleotide sequence used for de-multiplexing) on the 5’ end of the amplicon, which were followed by sequencing adapters. The mixture for outer PCR contained 0.2 μl of DNA polymerase (5 U/μl), 2.5 μl of 10× buffer A (KAPA Taq PCR Kit, Kapa Biosystems, USA) together with 1 μl of 20× EvaGreen™ (Biotium, Fremont, California, USA), 0.5 μl of 10 μM dNTP solution, ~ 20 ng of nucleic acid from the primary PCR and 3 μl of 10 μM primer solution in a total volume of 20 μl. This secondary PCR run was performed for 8 cycles including denaturation at 95 °C for 10 s, annealing at 60 °C for 10 s and 40 s elongation at 65 °C with 3 min final elongation at 72 °C. The quality of the amplification products was visualized by electrophoresis on 1.5% agarose gel after each amplification run. Based on this analysis, some amplicons were discarded due either to an unsuccessful PCR or out of range size.

The size range and quantity of nucleic acid in each individual library were assessed using a DNA 1000 kit on an Agilent Bioanalyzer 2100 (Agilent Technologies, Santa Clara, California, USA) (see Additional file 5). Prior to sequencing, all suitable amplicons were pooled together at equimolar quantities, then purified with E-Gel® SizeSelect™ (Invitrogen, Carlsbad, California, USA) and quantified with a Qubit 2.0 fluorimeter (Thermo Fisher Scientific, Waltham, Massachusetts, USA). Amplicon libraries were concentrated with Omega Cycle Pure Kit (VWR International, Radnor, Pennsylvania, USA) according to the manufacturers’ instructions and the subsequent DNA quantification was performed with a Qubit dsDNA Assay Kit (Molecular Probes, Eugene, Oregon, USA) on a Qubit 2.0 fluorimeter (Thermo Fisher Scientific, Waltham, Massachusetts, USA).

Sequencing was carried out on an Ion Torrent PGM™ sequencing platform (Thermo Fisher Scientific, Waltham, Massachusetts, USA) and the library prepared using Ion PGM Hi-Q View OT2 Kit (Thermo Fisher Scientific, Waltham, Massachusetts, USA) according to the manufacturers’ instructions. The library was loaded on an Ion 314 chip (Life Technologies, Carlsbad, California, USA), and sequenced for 800 cycles using an Ion PGM Hi-Q View Sequencing Kit (Thermo Fisher Scientific, Waltham, Massachusetts, USA).

### Post-sequencing analysis

Raw sequencing data from PGM runs were imported into CLC Genomics Workbench 10.1.1 (Qiagen, Hilden, Germany) to perform trimming. Briefly, low quality (trimming limit = 0.05) and ambiguous nucleotides, adapters and short residual reads (< 100 nucleotides) were removed. Filtered reads were then assembled into contigs with an overlap-layout-consensus (OLC) approach. The original reads were re-mapped on the assembled contigs to allow visual inspection of the correctness of the assembly of each contig. Sequences supported by less than 3 reads were discarded without further analysis and in some cases contigs displaying a high amount of polymorphism were re-assembled with more stringent parameters to obtain all the possible sequence variants. Transcripts were blasted (using BLASTx) against a custom sequence database containing all known Class-1 (Ranidae and Hylidae) or Class-3 (Bombinatoridae) nucleotide sequences to detect all positive hits based on E-value threshold of 0.05. The longest ORFs for each of the resulting contigs were translated into amino acid sequences using the ExPASy translate tool [26] and grouped together based on common features.

### Peptide characterization

Two peptides with amidated C-terminus were obtained from GenicBio Ltd. (Shanghai, China) at > 98% purity as confirmed by RP-HPLC and MS. They were dissolved in doubly distilled water and stock concentration determined as described previously [27, 28]. Minimal inhibitory concentration (MIC) was determined on a Gram-negative and a Gram positive reference laboratory strains, *Escherichia coli* ATCC 25922 and *Staphylococcus aureus* ATCC 29213, obtained from the American Type Culture Collection (ATCC, Rockville, MD, USA), using the serial two-fold microdilution method as described previously [27]. Cytotoxic effects on metabolic activity were determined on human monocytes isolated from buffy coats of informed donors (in accordance with the ethical guidelines and approved from the ethical committee of the University of Trieste) [29], using the 3-(4,5-dimethylthiazol-2-yl)-2,5-diphenyltetrazolium bromide (MTT) assay after treatment with the peptides for 24 h, as described previously [30].

## Results

### Novel peptide sequences

Selective amplification with AMP specific primers of cDNA libraries obtained from seven anuran species (Ranidae, Hylidae, Bombinatoridae families), followed by long-read high-throughput sequencing, resulted in 120.371 reads. Out of the assembled contigs, 5–15% of the sequences from the Ranidae family corresponded to AMPs, depending on the species. The success rate for *Hyla arborea* (1 contig, 0.2%) was much lower (see Table 2). *Bombina variegata* gave no contigs corresponding to AMPs, while *Bombina bombina* amplicons were not pooled with the others for sequencing, due to their excessive average size (573 nucleotides), as evaluated by Agilent Bioanalyzer 2100. This was suggested by the DNA fragment size restrictions (< 450) of the Ion Torrent™ PGM sequencer that was used.

**Table 2:** Number of raw and trimmed reads, assembled contigs and putative antimicrobial peptides classified per species and the primer of choice

Species	Forward primer	Raw reads	Trimmed reads	Contigs	AMPs (on target)
*Pelophylax* kl. *esculentus*	TP1	10.278	7.674	399	22 (5%)
	TP2	6.995	3.786		
	TP3	18.067	13.321		
*Pelophylax ridibundus*	TP1	5.401	4.192	196	31 (15%)
	TP2	3.670	2.446		
	TP3	5.132	3.924		
*Rana arvalis*	TP1	8.292	6.294	391	31 (8%)
	TP2	12.498	6.272		
	TP3	1.505	1.101		
*Rana dalmatina*	TP1	4.404	2.697	275	20 (7%)
	TP3	17.884	9.748		
*Rana temporaria*	TP1	6.751	4.164	187	23 (12%)
*Hyla arborea*	TP4	11.161	4.690	448	1 (0.2%)
*Bombina variegata*	TP5	8.333	4.807	577	0

Overall, this approach permitted to identify 128 likely AMP sequences. One hundred twenty seven of these were identified in the five species from the Ranidae family, and only one in *Hyla arborea*, from the Hylidae family (see Table 2). Before proceeding further with the analysis, we extracted the putative mature peptide region by removing the sequence N-terminal to the basic Lys-Arg propeptide cleavage site. The predicted peptides were then checked for identity with known sequences using BLASTp [31] and manually grouped into eight different classes. Seven of these classes were grouped based on the predicted secondary structure (e.g. α-helix, β-sheet), length, frequency of specific amino acids (e.g. presence of a characteristic rana-box domain) [32] and identity with known AMP classes. The eight class consisted of peptides which could not be classified into any of the other seven groups (see Additional file 6). About half of the peptides had already been identified previously in the same, or in closely related, species (48% with 100% identity), or shared significant similarity with previously described AMPs (27% with 81–99% identity). Thirteen of the peptides (10% of the total) showed significant BLAST e-values but < 80% identity with AMPs deposited in the protein sequences databases (see Table 3 and Additional file 7). Additionally, we could identify 16 entirely novel peptides (12%), here defined as peptides lacking significant similarity with known AMPs, but that may have antimicrobial activities based on their physico-chemical characteristics (charge, overall hydrophobicity) and the conserved secretion signal sequence (see Table 3 and Additional file 7). Four additional peptides (3%) with 100% identity to known peptide sequences were also identified. However, with less than 70% query cover and additional amino acids in their primary structure those were also categorized as novel (see Table 3 and Additional file 7). It is worth noting that some of these peptides were found to be identical (at the amino acid level) in multiple analyzed species, thereby reducing the number of completely unique novel peptide sequences to 14, and to 11 for peptides with less than 80% identity. Altogether, these results validate the reliability of this approach, confirming the effectiveness of the method that has been proven to be fast, accurate and suitable for simultaneous identification of large numbers of AMP sequences from Ranidae. Some potential limitations are discussed in the following section.

**Table 3:** Identified putative AMP sequences with no significant identity to the known proteins, less than 80% identity to known AMP sequences or 100% identity to proteins stored at NCBI database with < 70% query cover and their physico-chemical characteristics

#	Sequence	Species	Charge	H^a^
No identity to proteins stored at NCBI nr database				
1	FLGALGNALSRVLGK	*R. temporaria*	+3	0.813
2	FIGALVNALTRVLGK	*R. temporaria*	+3	1.213
3	FIGALVHALTGILGK	*R. temporaria*	+ 2	2.247
4	LVPFIGRTLGGLLARFGK	*R. temporaria*	+ 4	1.500
5	VPQLCFKFQKVIYCEINRTLPNEA	*R. dalmatina*	+ 2	−0.625
6	FSQLFFAWLLRLCRQ	*P.* kl. *esculentus*	+3	2.587
7	GIVEAWPLR	*P. ridibundus*, *R. dalmatina*	+ 1	1.133
8	NNLRHIVAWCKNRNYSLAVCARFKPQ	*R. temporaria*	+ 6	−1.612
9	NLLGFLQGAKDILKECEADNYQGWLCESYKPQ	*R. dalmatina*	−1	−1.250
10	FLPLVLGKTHSEQAEILSWKSSNVEYHLPKCTTDV	*P. ridibundus*, *R. dalmatina*	0	−0.763
11	FLPLIAGLWVNCSANNPKMLKLWK	*P.* kl. *esculentus*	+ 4	1.363
12	FLPICDKSALRFVGKV	*P. ridibundus*	+3	0.494
13	EMPMKKKEETIQKKGMLKWKTIFTSHCWSFE	*P. ridibundus*	+ 4	−1.835
14	RGLLDPITGLVGGLLR	*H. arborea*	+ 2	1.213
< 80% identity to proteins stored at NCBI database				
15	FLGFVGQALNALLGKLGK	*R. dalmatina*	+3	1.550
16	FLPAIAGILSQIFGK	*P.* kl. *esculentus*	+ 2	2.540
17	FFPAFLKVAAKVVPSIICSITKNVET	*P.* kl. *esculentus*	+3	0.573
18	IVPILLGVVPQLVCAITKKC	*R. dalmatina*, *R. temporaria*	+3	1.675
19	IIPLLLGKVVCAITKKC	*R. dalmatina*	+ 4	1.271
20	LVPMFLSKLICFITKKC	*R. temporaria*	+ 4	1.918
21	GLEVLGKILSGILGK	*R. dalmatina*, *Rana arvalis*	+ 2	1.220
22	LLGAALSALSSVIPSVISWFQKG	*Rana arvalis*	+ 2	1.670
23	LANRAARNTSQNVLNAITCTL	*R. dalmatina*	+3	−1.662
24	ADFLDKLRNFAAKNLQNKASL	*P. ridibundus*	+3	−1.595
25	EMLRKKEETIQKKGMLKWKNDFYQSLLEF	*P. ridibundus*	+3	−1.779
100% identity to proteins stored at NCBI database with < 70% query cover				
26	QKTYNRRPPGWSLYVFHQQISNLELEVI	*P.* kl. *esculentus*	+ 2	−0.654
27	FVPLLVSKLVCVVTKNVRIWKLELEII	*Rana arvalis*	+3	1.663
28	FVPLLVSKLVCVVTKNVRTLET	*Rana arvalis*	+3	0.455
29	FLPIVTNLLLRFVG	*R. dalmatina*	+ 2	3.729

^a^Calculated using the CCS consensus hydrophobicity scale [50]

### Preliminary biological characterization

Two of the novel identified peptides were synthesized (see Table 4) and tested for their in vitro activity against a Gram-negative and a Gram-positive reference bacterial strains. Results are promising, with both peptides active against *S. aureus*, especially rarv_10.1_19 with MIC of 4 μM. On the other hand, peptides don’t seem to be selective for Gram-negative strains (see Table 4) with MIC > 64 μM. Another encouraging aspect was the very low toxicity towards human circulating blood cells, namely monocytes. At the highest concentration used (100 μM) over 80% of cells were fully viable (see Table 4).

**Table 4:** Peptides tested for biological activity and their physico-chemical characteristics

Peptide^a^	Coding sequence	Sequence^b^	MW	Charge	MIC (μM)^c^		%Viability^d^ (100 μM)
					*E. coli*	*S. aureus*	Monocytes
#4	rtemp_4.3_210	LVPFIGRTLGGLLARF-NH2	1729.1	+ 4	> 64	4	> 80
#22	rarv_10.1_19	LLGAALSALSSVIPSVISWFQK-NH_2_	2286.7	+3	> 64	16	> 80

^a^Peptide number refers to designation in Table 3

^b^Sequences end with a C-terminal amidation signal [51, 52]

^c^Evaluated using microdilution assay in MH medium

^d^Viability assessed in the presence of 100 μM peptide after 24 h exposure by the MTT assay

## Discussion

Selective amplicon sequencing resulted in the simultaneous identification of antimicrobial peptide encoding transcripts from 5 out of 7 different anuran species tested. All the species with a positive result pertain to the Ranidae family, with a single sequence obtained from *Hyla arborea* (Hylidae) and none from *Bombina variegata* (Bombinatoridae) (see Table 2). The different success rate of this approach among three anuran families can be explained by several factors. First, the assembled transcriptome data used for primer design comprised several species pertaining to either the *Rana* or *Pelophylax* genus (Ranidae), whereas limited data with satisfactory phylogenetic relations was available for Hylidae. The high number of AMP transcripts available in the public databases for the two Ranidae genera enabled to construct three primers suitable for efficiently amplifying the different expressed AMP transcripts in the five target species, improving the chances of correct annealing and amplification in PCR. As the nucleotide sequence data available for other genera increases in the databases, it should become possible to refine primer design and provide suitable primer options also for other more distantly related families.

Another important parameter is transcript size. All known Class-1 Ranidae peptides are shorter than 100 AA long (mostly 70–80). Furthermore, our transcriptome screening revealed that the 3’ UTR region of the encoding transcripts was generally shorter than 200 nucleotides. The Ion Torrent™ PGM (Life Technologies, Carlsbad, California, USA) sequencing kit used is suitable for sequencing DNA strands up to about 450 base pairs. The design of degenerate primers based on the Class-1 signal peptide region should therefore have generated amplicons with a size range compatible with this sequencing method. This was confirmed using an Agilent Bioanalyzer 2100 prior to sequencing, which revealed a library size of between 300 and 400 bp for all the Ranidae species (see Additional file 5). This being said, libraries at the top of this size range, at around 400 bp, were very close to the maximal permissible size range, which could create problems. Amplicons successfully obtained from the *Bombina* species were excluded from sequencing for this reason, as their average size was > 500 nucleotides. Using the new isothermal amplification for Ion Torrent, or a different sequencing platform, may partly counteract this issue.

A third consideration comes from a detailed analysis of the data from Ranidae species. We obtained 22 unique assembled sequences in *P*. kl. *esculentus*, 31 in *P. ridibundus*, 31 in *R. arvalis*, 20 in *R. dalmatina* and 23 in *R. temporaria*. These results highlight the considerable sequence diversity of Class-1 AMPs in Ranidae. Identification of such a high number of variants within a single specimen underlines the fact that these AMPs are encoded by multigenic families [3]. Although the high conservation of the signal peptide region allowed targeted DNA sequencing, this feature can represent a potential obstacle in whole transcriptome sequencing. Indeed, the inefficient assembly of full length transcripts, or the collapse of similar variants within a single contig are well-known issues linked to the use of short reads in the assembly of highly similar transcripts derived from multigenic families [33, 34]. Therefore, we suggest the use of longer reads. In this respect, those obtained by Ion Torrent may represent an optimal balance between high-throughput and reasonable length (up to 450 bp) for the management of this sequence diversity. For the same reason, the use of three different primers is another key factor for successful amplification. This guarantees an efficient pairing with all the possible sequence variants. Indeed, even within the Ranidae family, we noticed substantial differences in the efficiency of the amplification using the 3 primers across species (see Additional file 8).

Considering that only one Class-1 AMP encoding transcript was obtained from *H. arborea* (Hylidae family), the explanation could thus be a combination of *i)* the poor representation of transcriptomic datasets from *Hyla* spp. in the SRA database; *ii)* the size range of the library, which was close to the maximum capabilities of Ion Torrent sequencing (see Additional file 5), and *iii)* a different AMP gene organization for this family. With respect to the first consideration, 4 out of 5 transcriptomes used for primer design pertained to a species of a different subfamily (Pelodryadinae) then *Hyla* (Hylinae) and the only transcriptome available for *Hyla arborea* was not obtained from AMP-producing tissues. It thus seems likely the designed forward primer did not include all the polymorphisms present in AMPs from the *Hyla* genus, resulting in the amplification of a single but highly represented sequence (22% of the total sequencing output). Concerning the second consideration, it is likely that AMP amplicons had been removed during the E-gel purification procedure, due to excessive size. The third consideration could be relevant if, unlike Ranidae family peptides, *Hyla* Class-1 AMPs do not have a multi-gene organization, even though this seems to be disproved by previous reports [3]. Based on these observations a similar approach should be undertaken in the future with an improved primer design, based on broader taxonomical sampling, specifically including other Hylinae species.

Unsuccessful results with Bombinatoridae are most certainly linked to the longer AMP precursor and, consequently, longer length of the encoding mRNA molecules. During the initial phases of the experimental design, we tried to overcome this issue by designing more internal primers based on the propeptide rather than on the signal peptide region, thereby reducing the size of the expected amplicons. However, the assessment of the library size range indicated that amplicons were above (*B. bombina*) or very close (*B. variegata*) to maximal input length capabilities of the sequencing technologies used. While the former library was discarded altogether, the latter one was subjected to sequencing but did not produce any positive matches. Despite a similar apparent size range between the libraries obtained from *B. variegata* and some of the longer ones obtained from Ranidae species, the concentration of the former was approximately 7 times lower (see Additional file 5). The most likely cause of unsuccessful sequencing in this species is therefore the removal of AMP amplicons during the E-gel purification procedure due to their borderline size, similarly to *H. arborea*, so that only non-specific amplicons were sequenced. To confirm this hypothesis we carried out purification of a single 573 bp band of *B. bombina* amplification visible on the agarose gel, followed by sequencing on a Sanger ABI 3130 sequencer (Thermo Fischer Scientific, Waltham, Massachusetts, USA). Although the chromatogram was not clean, suggesting that multiple products of the same size were amplified, the consensus sequence clearly confirmed the amplification of a Class-3 AMP precursor. Therefore, while our strategy was not suitable with Bombinatoridae for Ion Torrent PGM, other massive parallel sequencing platforms allowing higher read lengths (such as SMRT PacBio or Oxford Nanopore) could enable its application also in this anuran family.

Overall, the positive results obtained with Ranidae species, with the identification of 127 peptides, including several novel AMPs (i.e. lacking significant sequence similarity with previously characterized anuran sequences) confirm the effectiveness of this experimental design, as long as the degenerate primers are properly designed, and the amplicon size is tailored to the sequencing platform used. Geographical location would have been very important if the selected species were endemic to Croatia. However, in this case all the species targeted display a relatively broad and partially overlapping area of distribution across Europe and are, in some cases, evolutionarily closely related (e.g. the latest common ancestor of *R. dalmatina*, *R. temporaria* and *R. arvalis* lived in the Miocene [35]). Consequently, the expansion of the taxonomical breadth of sampling to other species adapted to different geographical locations, environmental niches and thereby evolving under different microbial contexts might represent a reliable strategy for novel anuran AMP discovery.

The single result obtained from *H. arborea* indicates that the panel of species analyzed can be quite wide, but this requires a particular effort in collecting as many sequences as possible from species phylogenetically closely related to the target species in the analysis panel to optimize primer design, thereby maximizing the chances of annealing during selective amplification. In this respect, PCR experimental conditions, and the annealing temperature in particular, need to be carefully selected to allow pairing with templates that are not perfectly matching, and allow capturing of as many sequence variants as possible. The lack of success in obtaining Bombinatoridae AMPs instead pinpoints the importance of tailoring the sequencing platform to the expected amplicon length, or alternatively, to identify useable sequences and design primers as close as possible to 3′ end of the mRNAs in order to reduce the amplicon size.

Although the novel identified peptides display physico-chemical features compatible with antimicrobial activity (see Table 3) and clearly possess a well-conserved signal peptide/propeptide region typically found in anuran AMPs, their biological role requires confirmation. A comprehensive evaluation of the antimicrobial activity of six selected novel peptides identified in this study is currently in progress, and preliminary results are presented for two of these peptides **(**see Table 4**)**. This confirms the reliability of our approach to identify novel, functional antimicrobial peptide sequences (manuscript in preparation).

## Conclusions

The approach here presented, with suitable modifications, can be applied also to other gene families sharing a conserved signal peptide and/or propeptide region, an hypervariable mature peptide region, and a limited distance between this region and the mRNA poly(A) tail. These characteristics are well known in many different animal AMPs [36–41], toxins [42–44] and other types of bioactive peptides from other organisms [45]. One should however always keep in mind a key factor, i.e. the detection of sequence variants depends on their being expressed. Therefore, whenever possible, the most appropriate tissue and/or experimental challenge need to be selected to enhance the expression of the target mRNAs. In our case, the choice of anuran skin was amply supported by abundant literature [5, 46–49], and indeed we could obtain several dozens of different peptides for each species as expected. However, the number of reads obtained for each sequence variant does not necessarily depend only on the level of expression of the transcript itself but is also affected by the efficiency of the amplification, which depends on the match to the primer. For this reason, the results of this type of study can only be considered as qualitative, and not as a proxy to investigate the expression levels of AMP variants. Finally, the small amount of tissue required may permit the identification of novel AMPs from endangered species, potentially without the need for sacrificing any individual. This would permit to fully exploit animal biodiversity in identification of potential novel therapeutic agents, without adding to the threat of reducing it.

## Additional files

Additional file 1: Complete list of frog species obtained from Croatian wild. (DOCX 13 kb)

Additional file 2: List of transcriptome data downloaded from SRA database. (DOCX 14 kb)

Additional file 3: List of signal peptides used for transcriptome screening. (XLSX 46 kb)

Additional file 4: Clusters of nucleotide alignments used for forward primer design. (DOCX 7338 kb)

Additional file 5: Size range of each individual library obtained prior to pooling. (DOCX 14 kb)

Additional file 6: Classification of identified peptides. (DOCX 1600 kb)

Additional file 7: BLASTp output for novel identified putative AMP sequences. (XLSX 47 kb)

Additional file 8: Success rate of amplicon synthesis in Ranidae species based on used primer. (DOCX 13 kb)

## Abbreviations

AMP: Antimicrobial peptide
OLC: Overlap layout consensus
ORF: Open reading frame
RACE: Rapid amplification of cDNA ends
