# Supplementary material for: Parallel identification of novel antimicrobial peptide sequences from multiple anuran species by targeted DNA sequencing
Source: BMC Genomics. 2018 Nov 20;19:827. doi: 10.1186/s12864-018-5225-5 (PMC6245896; doi:10.1186/s12864-018-5225-5)
Supplement: Supplementary file 1 — Complete list of frog species obtained from Croatian wild. (DOCX 13 kb) [file 12864_2018_5225_MOESM1_ESM.docx]

**Additional file 1.** Complete list of frog species obtained from Croatian wild, together with date and location of capture.

| # | | Family | | Species | | Date/location | | Coordinates | |
| --- | --- | --- | --- | --- | --- | --- | --- | --- | --- |
| 1 | | Ranidae | | *Pelophylax ridibundus* | | 8 April 2017 / Sutina river canyon | | E00507564 N04842600 377m | |
| 2 | |  | | *Rana dalmatina* | | 23 April 2017 / Sutina river canyon | | E00504296 N04840136 558m | |
| 3 | |  | | *Rana arvalis* | | 11 March 2017/ Turopoljski lug, Turopolje | | E00476470 N05057003 98m | |
| 4 | |  | | *Rana temporaria* | | 11 March 2017/ Turopoljski lug, Turopolje | | E00476470 N05057003 98m | |
| 5 | |  | | *Pelophylax* kl. *esculentus* | | 11 March 2017/ Turopoljski lug, Turopolje | | E00476470 N05057003 98m | |
| 6 | | Hylidae | | *Hyla arborea* | | 24 March 2017 / Ivanić grad, Žutica forest | | E00496319 N05054211 97m | |
| 7 | | Bombinatoridae | | *Bombina variegata* | | 8 April 2017 / Sutina river canyon | | E00504131 N04840053 580m | |
| 8 | |  | | *Bombina bombina* | | 24 March 2017 / Ivanić grad, Žutica forest | | E00496319 N05054211 97m | |
