# Supplementary material for: Parallel identification of novel antimicrobial peptide sequences from multiple anuran species by targeted DNA sequencing
Source: BMC Genomics. 2018 Nov 20;19:827. doi: 10.1186/s12864-018-5225-5 (PMC6245896; doi:10.1186/s12864-018-5225-5)
Supplement: Supplementary file 2 — List of transcriptome data downloaded from SRA database. (DOCX 14 kb) [file 12864_2018_5225_MOESM2_ESM.docx]

**Additional file 2.** List of transcriptome data downloaded from Sequence Read Archive (SRA) database and assembled with Trinity 2.4.0.

| # | Family | Species | SRA ID | Tissue |
| --- | --- | --- | --- | --- |
| 1 | Ranidae | *Odorrana margaretae* | SRR3418419 | Skin |
| 2 |  | *Pelophylax lessonae* | SRR1199131 | Stomach |
| 3 |  | *Pelophylax nigromaculatus* | SRR1269553 | Brain gonad |
| 4 |  | *Pelophylax nigromaculatus* | SRR3418422 | Skin |
| 5 |  | *Rana chensinensis* | ERR069062 | Oviduct |
| 6 |  | *Rana pipiens* | SRR1185669 | Multiple organs |
| 7 |  | *Rana temporaria* | ERR914560 | Liver (exposure to ranavirus) |
| 8 |  | *Rana temporaria* | ERR914524 | Liver (exposure to *Batrachochytrium dendrobatidis*) |
| 9 | Hylidae | *Cyclorana australis* | SRR3901720 | Liver |
| 10 |  | *Hyla arborea* | SRR827704 | Multiple tissues |
| 11 |  | *Litoria rubella* | SRR3901722 | Liver |
| 12 |  | *Agalychnis callidryas* | SRR1561055 | Spleen |
| 13 |  | *Agalychnis callidryas* | SRR1560999 | Skin |
| 14 |  | *Cyclorana alboguttata* | SRR619481 | Muscle |
| 15 |  | *Cyclorana alboguttata* | SRR619476 | Muscle |
| 16 | Bombinatoridae | *Bombina orientalis* | ERR632225 | Liver |
| 17 |  | *Bombina bombina* | ERR632222 | Liver |
| 18 |  | *Bombina variegata scabra* | ERR632224 | Liver |
| 19 |  | *Bombina variegata variegata* | ERR632223 | Liver |
| 20 |  | *Bombina maxima* | SRR566659 | Blood |
| 21 |  | *Bombina maxima* | SRR566619 | Skin |
