# Supplementary material for: Parallel identification of novel antimicrobial peptide sequences from multiple anuran species by targeted DNA sequencing
Source: BMC Genomics. 2018 Nov 20;19:827. doi: 10.1186/s12864-018-5225-5 (PMC6245896; doi:10.1186/s12864-018-5225-5)

**Additional file 4.** Clusters of nucleotide alignments used for forward primer design. The name of each nucleotide set which was used for primer design is equivalent to the name of the corresponding primer.


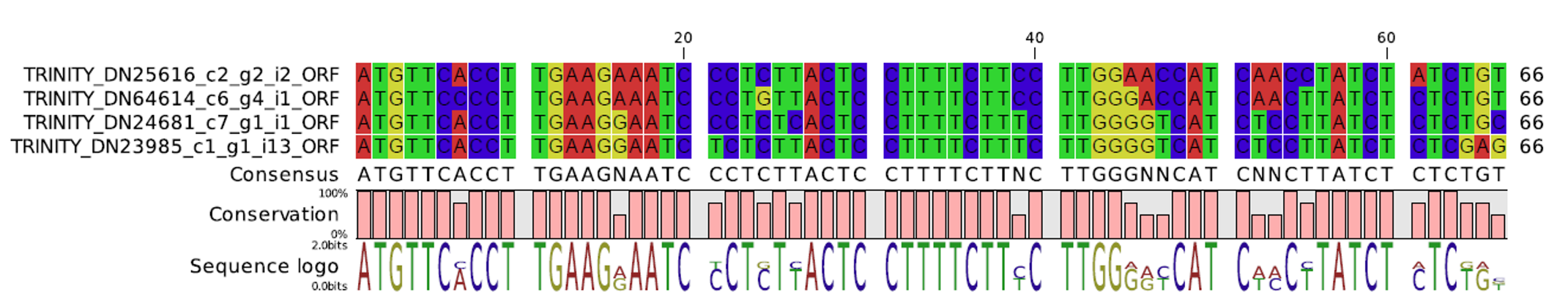
TP1


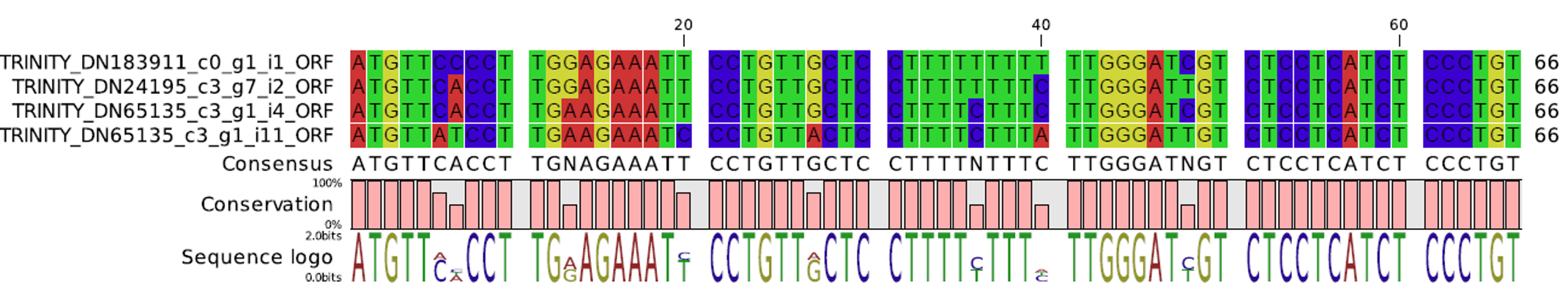
TP2


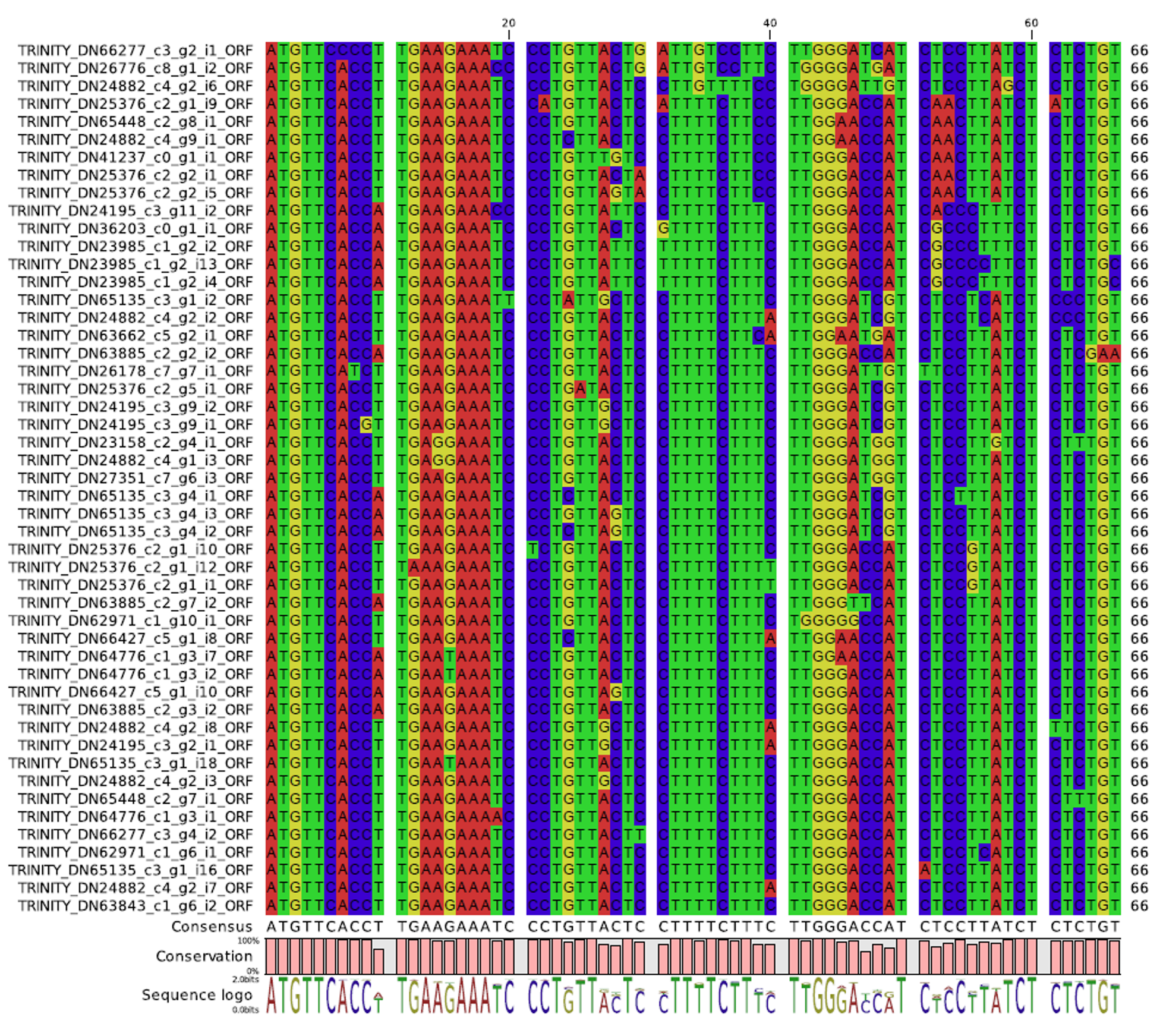
TP3


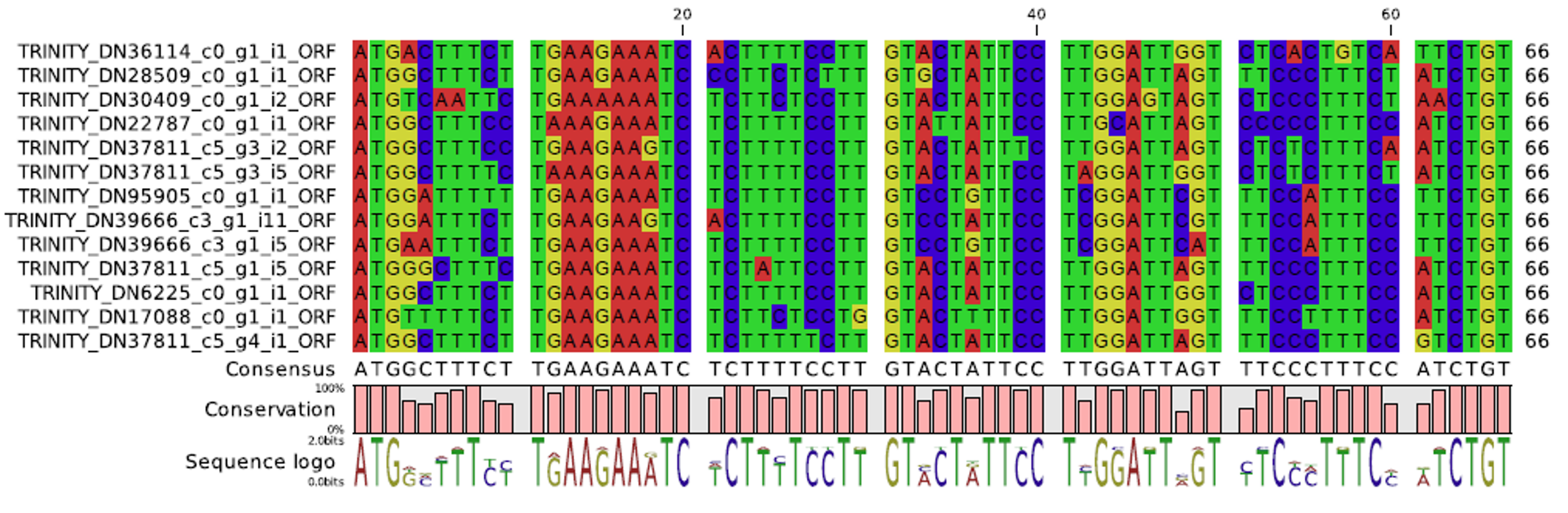
TP4

TP5


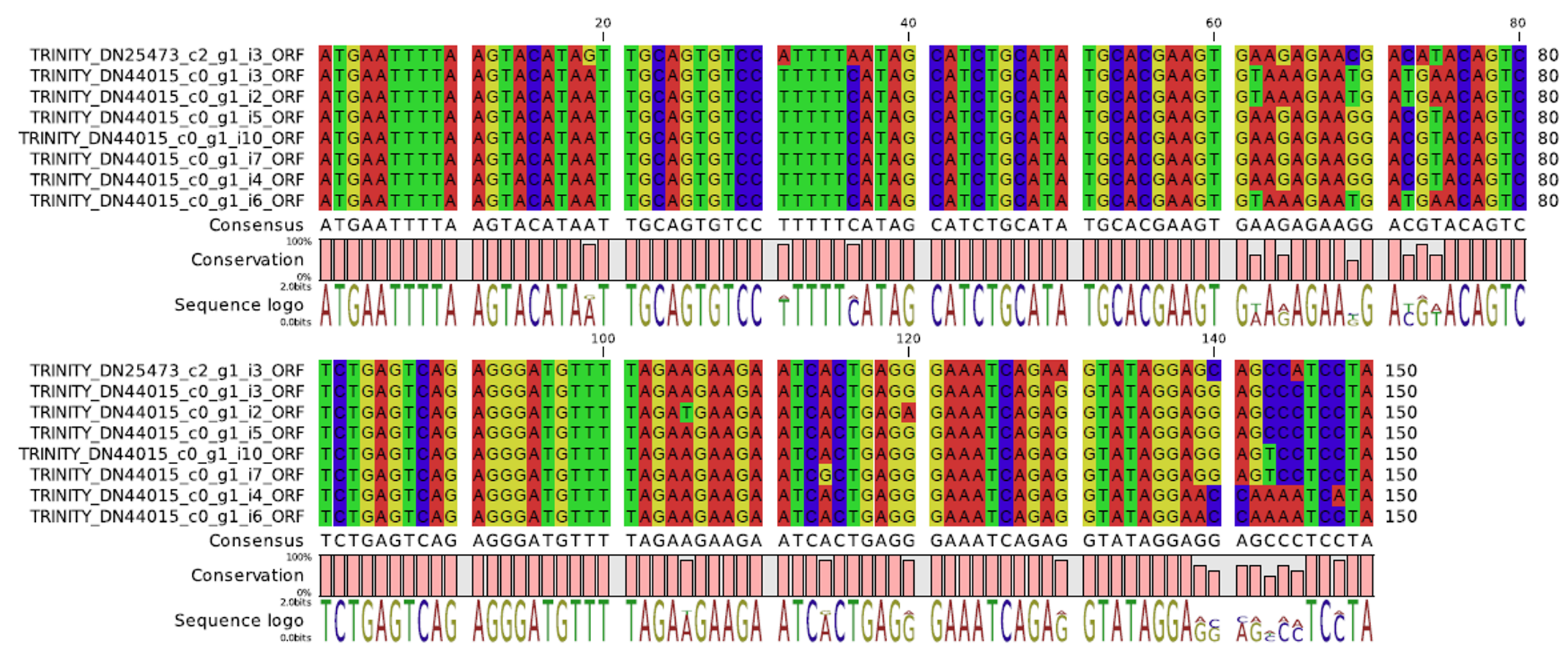

Supplement: Supplementary file 4 — Clusters of nucleotide alignments used for forward primer design. (DOCX 7338 kb) [file 12864_2018_5225_MOESM4_ESM.docx]
