# Supplementary material for: Parallel identification of novel antimicrobial peptide sequences from multiple anuran species by targeted DNA sequencing
Source: BMC Genomics. 2018 Nov 20;19:827. doi: 10.1186/s12864-018-5225-5 (PMC6245896; doi:10.1186/s12864-018-5225-5)
Supplement: Supplementary file 5 — Size range of each individual library obtained prior to pooling. (DOCX 14 kb) [file 12864_2018_5225_MOESM5_ESM.docx]

**Additional file 5.** Size range of each individual library obtained on Agilent Bioanalyzer 2100 prior to pooling. Size of the samples marked in gray were estimated based on the results obtained by electrophoresis migration on 1.5 % agarose gel.

| # | Forward primer | Species | Conc (nmol/l) | Base pairs |
| --- | --- | --- | --- | --- |
| 1 | TP1 | *Pelophylax* kl. *esculentus* | 26.2 | 394 |
| 2 | TP1 | *Pelophylax ridibundus* | 22 | 386 |
| 3 | TP1 | *Rana arvalis* | 22 | Compared to #2 |
| 4 | TP1 | *Rana dalmatina* | 22 | Compared to #2 |
| 5 | TP1 | *Rana temporaria* | 22 | Compared to #2 |
| 6 | TP2 | *Pelophylax* kl. *esculentus* | 9.1 | 364 |
| 7 | TP2 | *Pelophylax ridibundus* | 21.6 | 329 |
| 8 | TP2 | *Rana arvalis* | 5.5 | 366 |
| 9 | TP3 | *Pelophylax* kl. *esculentus* | 12.5 | 393 |
| 10 | TP3 | *Pelophylax ridibundus* | 35.7 | 363 |
| 11 | TP3 | *Rana arvalis* | 6.9 | 367 |
| 12 | TP3 | *Rana dalmatina* | 5.0 - 10.0 | Compared to #13 |
| 13 | TP4 | *Hyla arborea* | 5.0 | 440 |
| 14 | TP5 | *Bombina bombina* | N/A | 539 |
| 15 | TP5 | *Bombina variegata* | 2.8 | 389 |
