# Supplementary material for: Parallel identification of novel antimicrobial peptide sequences from multiple anuran species by targeted DNA sequencing
Source: BMC Genomics. 2018 Nov 20;19:827. doi: 10.1186/s12864-018-5225-5 (PMC6245896; doi:10.1186/s12864-018-5225-5)

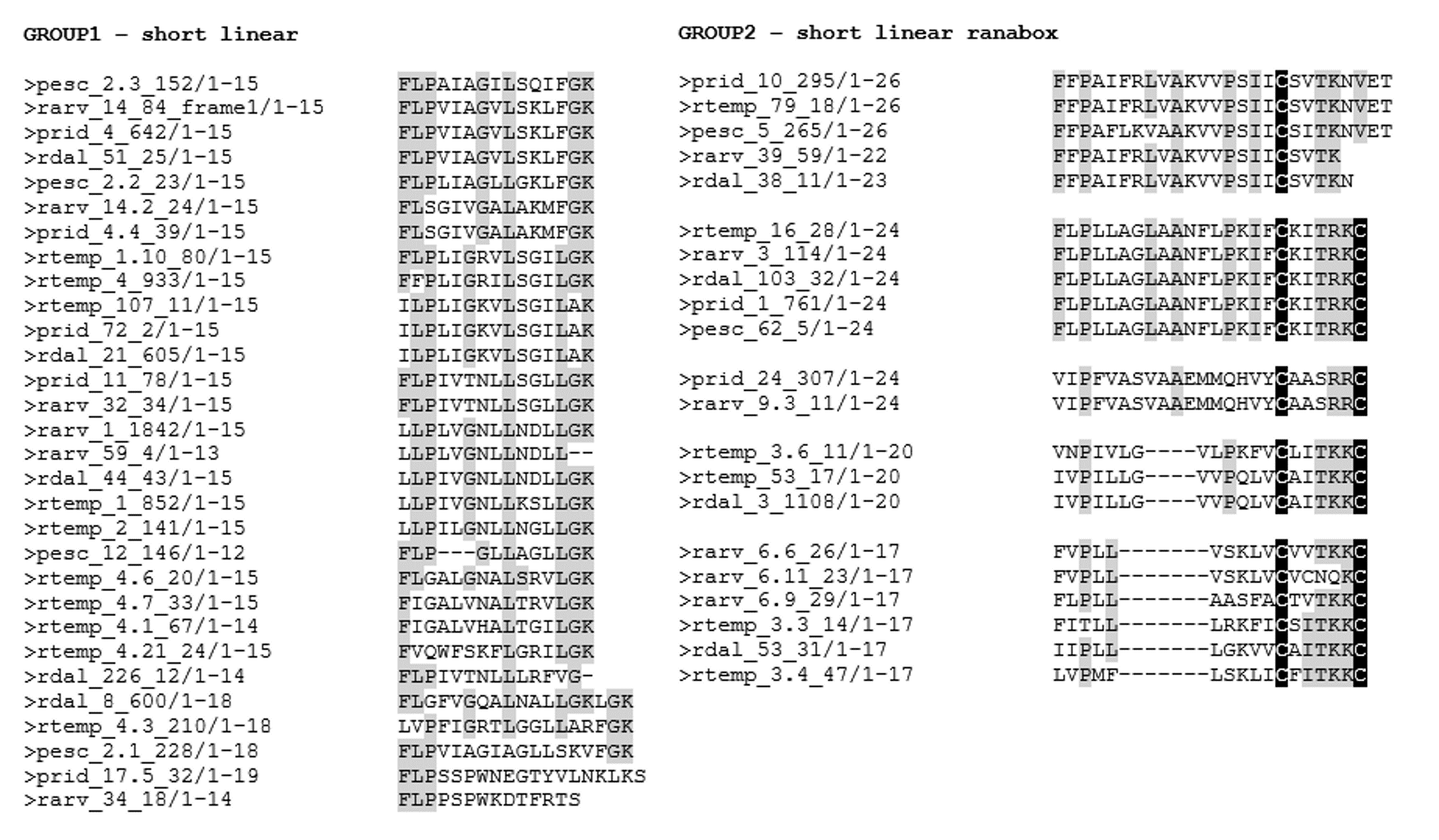
**Additional file 6.** Classification of peptides based on primary sequence properties and/or similarity to known protein sequences. Peptide name consists of the first letter of the frog genus where the peptide was identified, followed by 3 or 4 letters pertaining to the species name. First number refers to the contig where the peptide was found, and the second one to the number of reads supporting it. If the contig was re-assembled, it is indicated in the contig number (e.g. 4.3).


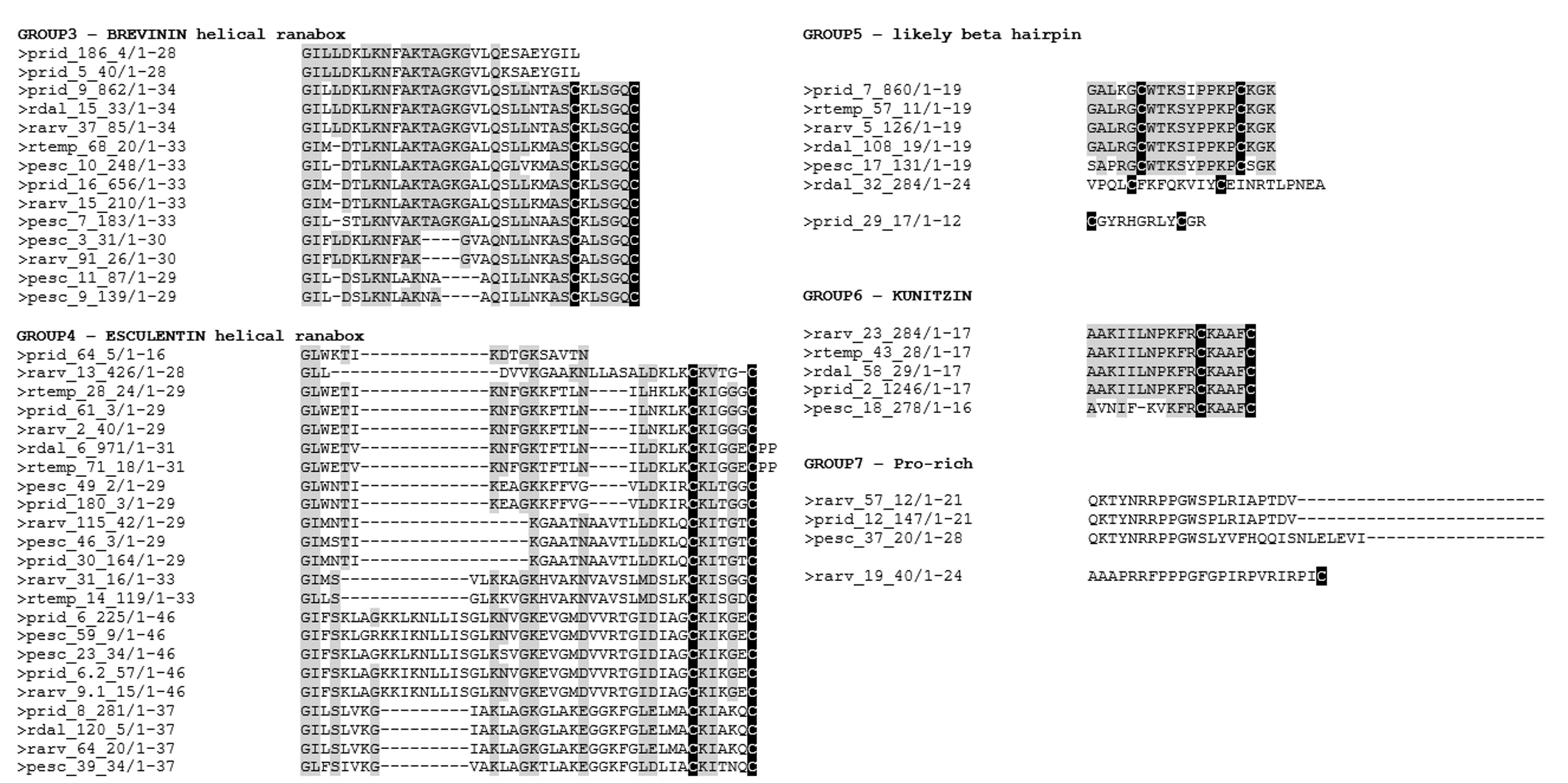


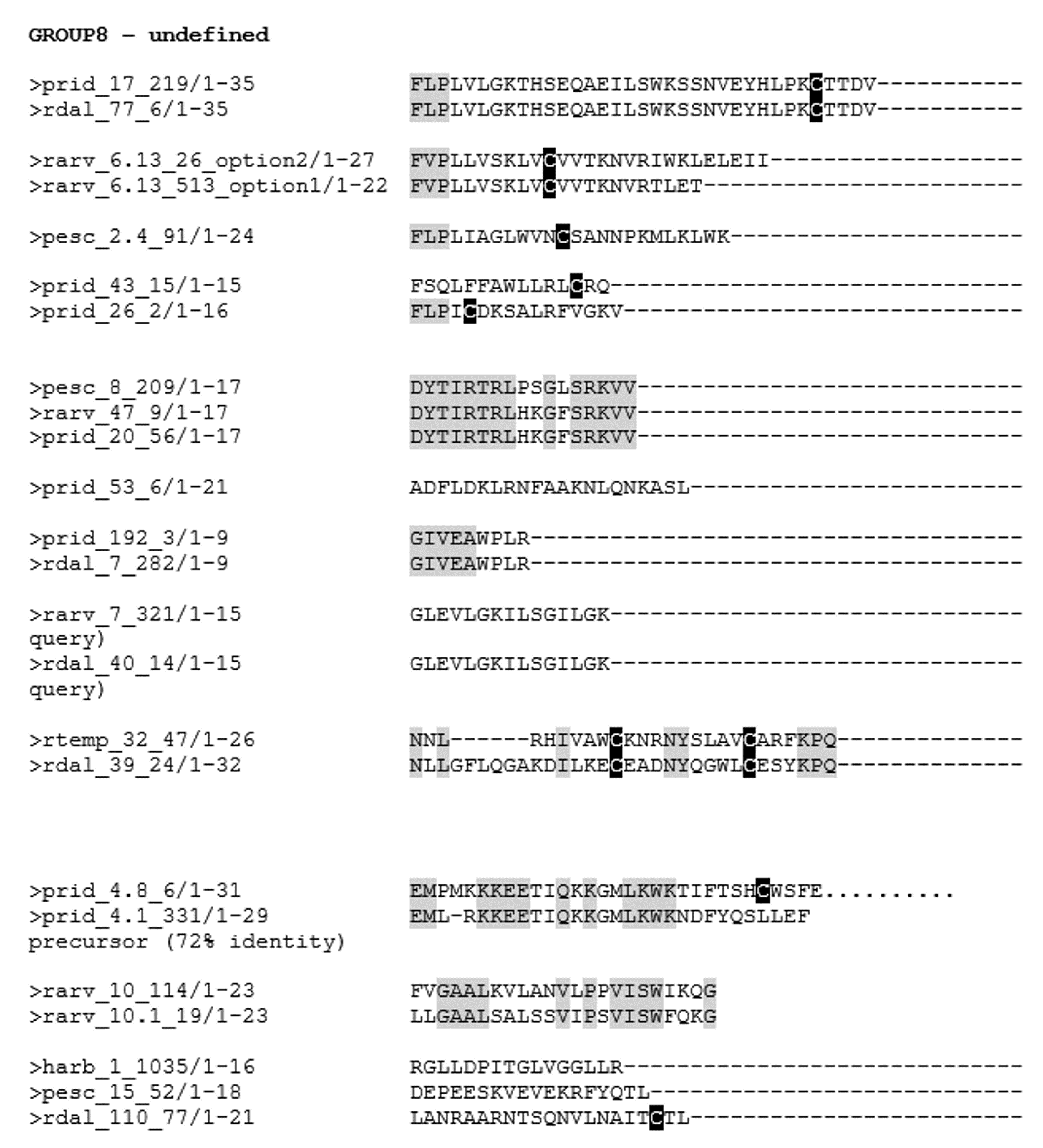

Supplement: Supplementary file 6 — Classification of identified peptides. (DOCX 1600 kb) [file 12864_2018_5225_MOESM6_ESM.docx]
