# Supplementary material for: Parallel identification of novel antimicrobial peptide sequences from multiple anuran species by targeted DNA sequencing
Source: BMC Genomics. 2018 Nov 20;19:827. doi: 10.1186/s12864-018-5225-5 (PMC6245896; doi:10.1186/s12864-018-5225-5)
Supplement: Supplementary file 8 — Success rate of amplicon synthesis in Ranidae species based on used primer. (DOCX 13 kb) [file 12864_2018_5225_MOESM8_ESM.docx]

**Additional file 7.** Success rate of amplicon synthesis in Ranidae species based on used primer (TP1, TP2 or TP3).

| Forward primer | Species | Amplification (Y/N) |
| --- | --- | --- |
| TP1 | *Pelophylax* kl. *esculentus* | Y |
|  | *Pelophylax ridibundus* | Y |
|  | *Rana arvalis* | Y |
|  | *Rana dalmatina* | Y |
|  | *Rana temporaria* | Y |
| TP2 | *Pelophylax* kl. *esculentus* | Y |
|  | *Pelophylax ridibundus* | Y |
|  | *Rana arvalis* | Y |
|  | *Rana dalmatina* | N |
|  | *Rana temporaria* | N |
| TP3 | *Pelophylax* kl. *esculentus* | Y |
|  | *Pelophylax ridibundus* | Y |
|  | *Rana arvalis* | Y |
|  | *Rana dalmatina* | Y |
|  | *Rana temporaria* | N |
